# The First Bioluminescence Tomography System for Simultaneous Acquisition of Multiview and Multispectral Data

**DOI:** 10.1155/IJBI/2006/58601

**Published:** 2006-12-04

**Authors:** Ge Wang, Haiou Shen, Kumar Durairaj, Xin Qian, Wenxiang Cong

**Affiliations:** Bioluminescence Tomography Laboratory, Department of Radiology, University of Iowa, 200 Hawkins Drive, Iowa City, IA 52242, USA

## Abstract

We describe the system design of the first bioluminescence tomography (BLT) system for parallel acquisition of multiple bioluminescent views around a mouse in a number of spectral channels simultaneously. The primary component of this BLT system is a novel mirror module and a unique mouse holder. The mirror module consists of a mounting plate and four mirrors with stages. These mirror stages are right triangular blocks symmetrically arranged and attached to the mounting plate such that the hypotenuse surfaces of the triangular blocks all make 45^∘^ to the plate surface. The cylindrical/polygonal mouse holder has semitransparent rainbow bands on its side surface for the acquisition of spectrally resolved data. Numerical studies and experiments are performed to demonstrate the feasibility of this system. It is shown that bioluminescent signals collected using our system can produce a similar BLT reconstruction quality while reducing the data acquisition time, as compared to the sequential data acquisition mode.


Hindawi Publishing Corporation
International Journal of Biomedical Imaging
Volume 2006, Article ID 58601, Pages 1–8
DOI 10.1155/IJBI/2006/58601

### The First Bioluminescence Tomography System for Simultaneous Acquisition of Multiview and Multispectral Data

**Ge Wang, Haiou Shen, Kumar Durairaj, Xin Qian, and Wenxiang Cong**

*Bioluminescence Tomography Laboratory, Department of Radiology, University of Iowa, 200 Hawkins Drive, Iowa City, IA 52242, USA*

Received 21 July 2006; Revised 30 August 2006; Accepted 5 September 2006

Recommended for Publication by Ming Jiang

We describe the system design of the first bioluminescence tomography (BLT) system for parallel acquisition of multiple bioluminescent views around a mouse in a number of spectral channels simultaneously. The primary component of this BLT system is a novel mirror module and a unique mouse holder. The mirror module consists of a mounting plate and four mirrors with stages. These mirror stages are right triangular blocks symmetrically arranged and attached to the mounting plate such that the hypotenuse surfaces of the triangular blocks all make $45^\circ$ to the plate surface. The cylindrical/polygonal mouse holder has semi-transparent rainbow bands on its side surface for the acquisition of spectrally resolved data. Numerical studies and experiments are performed to demonstrate the feasibility of this system. It is shown that bioluminescent signals collected using our system can produce a similar BLT reconstruction quality while reducing the data acquisition time, as compared to the sequential data acquisition mode.

Copyright © 2006 Ge Wang et al. This is an open access article distributed under the Creative Commons Attribution License, which permits unrestricted use, distribution, and reproduction in any medium, provided the original work is properly cited.

### 1. INTRODUCTION

Bioluminescent imaging has proven to be instrumental for studying gene expression, protein interaction, and cellular dynamics in small animal models, and promises to have major impacts on small animal studies towards the development of molecular medicine [1–3]. Bioluminescence tomography (BLT) is to reconstruct a bioluminescent source distribution inside a living mouse from optical signals measured on the body surface of the animal [4–20]. The BLT performance can be improved if the composite optical data are spectrally separated [14, 18, 20]. To collect multispectral bioluminescent data, optical filters are currently used one at a time in front of a CCD camera lens [18, 20]. To collect multiview images, the Xenogen IVIS™ bioluminescent imaging system 3D series utilizes a rotating mechanism to capture up to eight views one at a time [16]. Another system use a mirror system to collect four views of a mouse, but this system does not allow that the camera system is focused on all the four views, since these views have different distances to the CCD [18]. Both the systems are incapable of collecting multispectral data simultaneously [16, 18].

Since bioluminescent reporters are much dimmer than fluorescent reporters, bioluminescent data acquisition generally takes much longer time than that for fluorescent imaging, 5 to 10 minutes is normal for one exposure if the bioluminescence source is deep inside a mouse. The bioluminescent signal decays over about 1 hour. In most cases, we prefer taking four views (front, back, and two sides) around a mouse, one hour is adequate to take four pictures. However, there is an increasing interest in multispectral bioluminescence tomography, since multispectral images can provide much more information and realize the possibility to utilize more than one bioluminescence probes [21]. In the multispectral case, if we have four spectral channels, the one-hour window is not sufficient to take all the images sequentially. Hence, collecting multiview and multispectral bioluminescent signals simultaneously is extremely desirable for multispectral bioluminescence tomography.

In Section 2, we will describe the system architecture. In Section 3, we will present the multispectral BLT reconstruction method. In Section 4, we will evaluate the system performance in numerical and mouse studies using our multiview

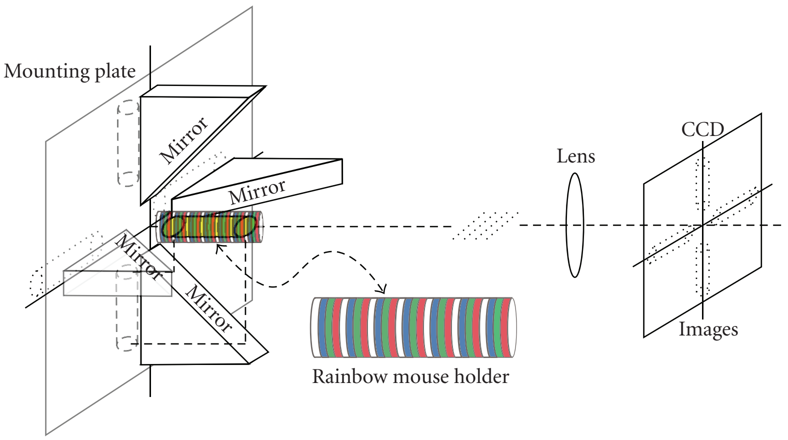

Figure 1: Bioluminescence tomography (BLT) system design for simultaneous acquisition of multiview and multispectral data. This system consists of a multimirror module, a cylindrical/polygonal mouse holder on which rainbow bands are attached for resolving data spectrally, a lens, and a CCD camera.

system. In Section 5, we will discuss relevant issues and conclude the paper.

### 2. SYSTEM DESIGN

There are currently four bioluminescence probes: hRLuc, CBGr68, Fluc+, and CBRed. The last two have spectral peaks in the orange/red wavelengths ($\sim$ 590 to 650 nm) [22]. In this range, the light is less absorbed with a greater penetration power through tissues. We can partition the spectral [500, 750] nm into three spectral ranges: [500, 590] nm, [590, 625] nm, and [625, 750] nm. The reason for this partition is that these intervals contain similar amounts of energy at the mouse body temperature 37°C for Fluc+ and CBRed [21, 22]. For Fluc+, the percentages are 28%, 33%, and 39% for [500, 590] nm, [590, 625] nm, and [625, 750] nm, respectively. For CBRed, the percentages are 14%, 35%, and 51%, respectively.

Our proposed BLT system design is mainly for parallel acquisition of multiview and multispectral bioluminescent data. Figure 1 shows the architecture of the system. It consists of a multiview mirror module, a mouse holder with patterned filter, and a CCD camera with a lens. In Figure 1, the multiview mirror module includes a mounting plate, four mirror stages, and four mirrors. The aluminum mounting plate is a square of 40 cm side length and 6 mm thickness. The aluminum mirror stage is right triangular blocks of 125 mm side length and 30 mm thickness. The triangular blocks are mounted on the mounting plate symmetrically around a mouse inside a transparent cylindrical (Syntec Technologies, Inc.) mouse holder of length 120 mm. A rectangular front mirror of size 160 mm$\times$30 mm is attached to each of the four hypotenuse surfaces of the mirror stages. Without use of the multispectral components, the mirror-based system can be a standalone multiview system. The four views of the mouse in the mirrors are parallel to the mounting plate surface. If the mouse is placed in the center of the four mirrors, the four views will be in the same object plane so that the camera can focus on all the four views simultaneously. To keep the mouse holder in position, it was attached to the mounting plate with a X-Y flexure stage, which can move the mouse holder as needed.

To enhance the multiview system into a multiview multispectral system, we employ a novel mouse holder, as shown in Figure 1. The mouse holder is a custom-designed transparent cylindrical (or polygonal) tube (Syntec Technologies, Inc.) of length 120 mm with interleaving filter bands that separate bioluminescent light into different spectral channels of interest. Several mouse holders with different radius (10 mm to 15 mm) will be employed in our system to match the size of the mouse. In our system, three different filters are placed on the side surface of the cylinder with 1 mm width for each filter band. One filter is a low-pass filter covering the spectral range [400, 590] nm, one filter is a band-pass filter for the range [590, 625] nm, and another filter is a high-pass filter for the range [625, 750] nm. The transmission at peak for each filter is greater than 80%.

This system uses a highly sensitive CCD camera (Princeton Instruments VersArray 1300 B, Roper Scientific, Trenton, NJ) which offers 1340 $\times$ 1300 pixels, 20 $\times$ 20 $\mu$m pixel size, and 16 bits dynamic range. From 500 nm to 750 nm, quantum efficiency (QE) is greater than 80%. With liquid nitrogen the camera can be cooled down to $-110$°C to make the dark current negligible. At this temperature, the typical CCD read noise is 2 e rms, and the dark current is less than 1 e/p/hr for 20 $\mu$m pixel. The camera is coupled with a Nikon 85 mm f/1.4 lens with about 27 $\times$ 27.8 cm field of view at 90 cm distance. To cover the 4 reflected bioluminescent views, the camera is placed 0.9 m away from the mounting plate to provide an adequate field of view. An absolute intensity calibration of the

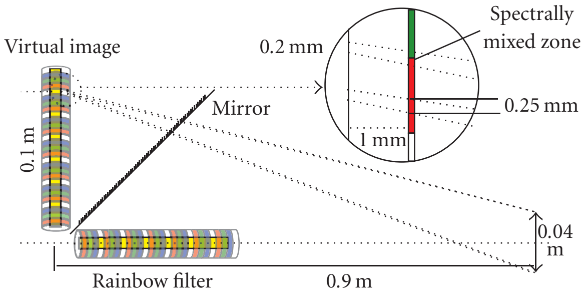

Figure 2: Zoomed view of light transmitting through the rainbow mouse holder.

CCD camera and the overall imaging system is necessary to recover the signal brightness in physical unit (W/cm$^2$/sr). For that purpose, the absolutely calibrated 8-inche-integrating sphere system from SphereOptics is used. A 4-inche sphere contains a tungsten lamp light source. A 6-position automated filter wheel with 5 filters (500 nm, 550 nm, 600 nm, 650 nm, 700 nm) and a variable attenuator with a dynamic range up to 11000 are placed between the two spheres to select a particular wavelength and control the light level entering the 8 inches sphere. The 2-inche output aperture of the 8-inche sphere allows as low as $2.07 \times 10^{-13}$ W/cm$^2$/sr in the spectral region of interest. By imaging this output aperture, the gray level of the CCD can be converted into physical unit. The camera is calibrated on f/1.4 and at the distance of 0.9 m. It turns out that each pixel gray level at the CCD is equivalent to $5 \times 10^5$ photons/sr/cm$^2$.

Note that not all pixels in the image can be used in the reconstruction. As shown in Figure 2, since the mouse and the mouse holder could not match perfectly. There is some distance between the mouse surface and the filters. As a result, some of the pixels contain light from two filters. We can select those pixels that only contain light from one filter around the central line of each filter band. As shown in Figure 2, with the size of the pixel $20 \times 20\,\mu$m, the corresponding area on the mouse is about $200 \times 200\,\mu$m. The distance between the mouse surface and the front lens is about 90 cm, while the size of the front lens is about 4 cm. Assume that the distance between the mouse surface and the filter is 1 mm, the size of each projected pixel on the filter is about 0.25 mm. Since each filter band is about 1 mm width, it can cover at least 3 pixels in width. From the above calculation, we can see that if the mouse holder is sufficiently tight, we can always find a good number of pixels recording only one spectral band information. The disadvantage of this approach is that some of the image pixels are wasted but they may be utilized using more sophisticated process and analysis, which is beyond the scope of this feasibility paper. A better way is to place a customized filter pattern just in front of the CCD chip or use an optical system to split light into different spectral bands and record the resultant signals. Since the mouse holder scheme is the easiest and cheapest, in this paper we focus on this idea to establish the feasibility of this multispectral bioluminescence tomography system design.

### 3. SIGNAL-TO-NOISE RATIO ESTIMATION

The signal-to-noise ratio (SNR) of a camera system can be computed as in [1]:

$$\mathrm{SNR} = \frac{S}{\sqrt{S + D \times t + N^2}}, \tag{1}$$

where $S$ is the signal per pixel in electrons, $t$ the integration time, $D$ the dark current value (electrons/pixel/second), and $N$ the CCD read noise (electrons rms/pixel). A typical way to increase SNR is by binning pixels prior readout. A binning value $k$ means that a group of $k \times k$ pixels is summed together to form one superpixel. By combining the pixels into one superpixel we make the signal and dark current $k^2$ times stronger than before. The readout noise remains the same for on-chip binning. The VersArray 1300 B CCD permits on-chip binning. Hence, we have the following result:

$$\mathrm{SNR}_{k\times k} = \frac{S \times k^2}{\sqrt{S \times k^2 + D \times t \times k^2 + N^2}}. \tag{2}$$

The trade-off of binning is spatial resolution. In our finite element algorithm for bioluminescence tomography, the size of each finite element is about 1 mm. In our system, each pixel is corresponding to $0.2\,\mathrm{mm} \times 0.2\,\mathrm{mm}$ on the mouse body surface. If we apply a single spectral algorithm, we can use $5 \times 5$ binning. If we apply a multispectral algorithm, we can use $3 \times 5$ binning, because along the width of each filter band two spectrally mixed pixels are discarded. For sequential systems, the field of view can be smaller to cover just one mouse view, which means they can use larger binning size to get better SNR given other conditions being identical.

There are two additional common ways to increase SNR: increasing signal strength, and increasing integration time. For bioluminescence imaging/tomography, we could not increase the strength of bioluminescent source but we can use a lens with bigger aperture (a customized f/1.0 lens can help) and employ a CCD with higher QE. As far as integration time is concerned, because we can capture multiview and multispectral images simultaneously, the integration time can be increased to improve the SNR.

In multispectral cases, only part of the energy can be utilized, the signal strength will be weaker than mix-spectral cases. Let S be the signal corresponding to spectral $[500, 750]$ nm, let $S_1$, $S_2$, and $S_3$ be the signal corresponding to spectral $[500, 590]$, $[590, 625]$, and $[625, 750]$ nm, respectively. We have $S = S_1 + S_2 + S_3$. The percentage of the signal in each spectral band depends on the probe, the optical coefficients of the tissues in different spectral bands, and the distance between a bioluminescent source to the mouse body surface. Generally speaking, in a short wavelength spectral band the signal is subject to more absorption and less scattering. Hence, if a light source is deep inside the tissue, spectral $[500, 590]$ nm can be very weak so that the CCD could not record a strong signal but it can still receive enough photons in the other two bands. In this case, we can still use multispectral data to do the reconstruction. Actually, the lack of photons in the spectral range $[500, 590]$ nm is informative for reconstruction, which tells us that the source must be relatively deep inside the animal.

### 4. NUMERICAL AND EXPERIMENTAL RESULTS

### 4.1. Multispectral bioluminescence tomography method

In bioluminescent imaging, photons from light-emitting luciferase probes inside a mouse may escape the mouse body subject to attenuation. In this process, photon scattering predominates over absorption. Hence, the photon propagation can be modeled as the well-known diffusion equation. Because the internal bioluminescent light is continuously on during the measurement, BLT operates only in the CW mode. Hence, we have the following steady-state diffusion equation and the Robin boundary condition [8–13, 21–24]:

$$-\nabla \cdot (D(\mathbf{r},\lambda)\nabla\Phi(\mathbf{r},\lambda)) + \mu_a(\mathbf{r},\lambda)\Phi(\mathbf{r},\lambda) = S(\mathbf{r},\lambda) \quad (\mathbf{r} \in \Omega),$$
$$\Phi(\mathbf{r},\lambda) + 2A(\mathbf{r},\lambda)D(\mathbf{r},\lambda)(\nu(\mathbf{r}) \cdot \nabla\Phi(\mathbf{r},\lambda)) = 0 \quad (\mathbf{r} \in \partial\Omega), \tag{3}$$

where $\Phi(\mathbf{r},\lambda)$ represents the photon fluence rate (W/mm$^2$) for spectral component $\lambda$ at location $\mathbf{r}$, $S(\mathbf{r},\lambda)$ the density of the bioluminescent source distribution (W/mm$^3$), $\mu_a(\mathbf{r},\lambda)$ the absorption coefficient (mm$^{-1}$), $\mu_s'(\mathbf{r},\lambda)$ the reduced scattering coefficient (mm$^{-1}$), $D(\mathbf{r},\lambda) = (3(\mu_a(\mathbf{r},\lambda)+\mu_s'(\mathbf{r},\lambda)))^{-1}$ the diffusion coefficient, $\Omega$ the support for the mouse body, $\partial\Omega$ the body surface, and $A(\mathbf{r},\lambda)$ the mismatch coefficient due to different refractive indices across $\partial\Omega$. With an adequate exposure time, a significant amount of bioluminescent photons can come out of the mouse and can be detected with the CCD camera. The measured quantity is the photon current on the body surface [21–24]:

$$\tilde{\Phi}(\mathbf{r},\lambda) = -D(\mathbf{r},\lambda)(\nu \cdot \nabla\Phi(\mathbf{r},\lambda)) \quad (\mathbf{r} \in \partial\Omega), \tag{4}$$

where $\nu$ denotes the unit outer normal to $\partial\Omega$. Based on (3)-(4), a linear system linking a bioluminescent source distribution and the boundary measurement can be briefly expressed as

$$\mathbf{B}(\lambda)\mathbf{S}(\lambda)=\tilde{\mathbf{\Phi}}(\lambda), \tag{5}$$

where $\mathbf{S}(\lambda) = \{S_1, S_2, \dots, S_M\}^T$ represents the discretized bioluminescent source distribution, $\mathbf{B}(\lambda) = \{\mathbf{b}^1, \mathbf{b}^2, \dots, \mathbf{b}^M\}$ a weighting matrix consisting of $N$-dimensional column vectors $b^k$ $(k = 1, 2, \dots, M)$, and $\tilde{\mathbf{\Phi}}(\lambda)$ an $N$-dimensional vector for the data measured on the body surface. Clearly, $S_k\mathbf{b}^k$ is the contribution of the $k$th source component $S_k$ to $\tilde{\mathbf{\Phi}}(\lambda)$. Then, the BLT problem is to reconstruct $S_i$ $(i = 1, 2, \dots, M)$ from the data $\tilde{\mathbf{\Phi}}(\lambda)$. This is a typical underdetermined problem. As a result, a strong prior knowledge must be incorporated into the reconstruction to overcome the ill-posedness effectively. Here we propose a multiscale BLT reconstruction procedure to refine permissible source regions gradually and improve image resolution accordingly. Initially, low resolution BLT can reliably identify clusters of bioluminescent sources. As image resolution of reconstructed bioluminescence sources becomes higher using an iterative procedure, permissible source regions can be better delimited with each iteration. Mathematically, the BLT reconstruction can be converted into the following optimization subject to regularization [22]:

$$\min_{\substack{0 \le S \le U \\ S \in \Omega_S}} \left( \sum_{\lambda} ||\mathbf{B}(\lambda)\mathbf{S}(\lambda) - \tilde{\mathbf{\Phi}}(\lambda)||_W^2 + \varepsilon\eta(S) \right), \tag{6}$$

where $U$ denotes the upper bound for the source to be physically meaningful, $\Omega_S$ a permissible source domain, $\eta$ a stabilizing function, $\varepsilon$ a regularization parameter, $W$ a weighting matrix, and $\| V\|_W^2 = V^T W V$.

### 4.2. Simulation results

We evaluated the system performance in numerical simulation using a heterogeneous cylindrical mouse phantom. This numerical phantom of diameter 20 mm and height 20 mm was designed to mimic the thoracic cavity of a mouse. The phantom contains four types of materials representing muscle (M), lungs (L), heart (H), and bone (B), respectively, as shown in Figure 3. The appropriate optical parameters were assigned to each of the four components as summarized in Table 1, which were extracted from the literature [17, 25–27]. The phantom was discretized into 36 000 wedge elements and 19761 nodes. On the side surface of the phantom, 1680 sampling locations were assumed along a number of virtual rings at different elevations. Each ring consisted of 80 sampling locations with sampling distance 0.78 mm. The distance between the adjacent rings was set to 1 mm for consistence with the interband distance on the rainbow holder. These rings were in correspondence to the spectral components.

Two bioluminescent sources were embedded in the lungs of the phantom at $(-2.7, -0.28, 10)$ and $(3.26, -2.52, 10)$, respectively, as shown in Figure 3. Based on the bioluminescent emission spectrum, three spectral bands were defined as $[500, 590]$ nm, $[590, 630]$ nm, and $[630, 750]$ nm. Each source had 6.0 nW with the spectral distribution 28% for $[500, 590]$ nm, 33% for $[590, 625]$ nm, and 39% for $[625, 750]$ nm. For each sequential image, we assume 3.5 minutes integrating time (total is 56 minutes for 16 images); and for our multiview and multispectral system, we assume 20 minutes integrating time. A finite element based forward solver in Matlab was employed to solve the forward problem on an Intel dual Xeon 2.8 GHz 2 GB memory Dell Dimension 670 machine. Then, we converted the phantom side surface photon fluence from floating point W/cm$^2$ into photon/cm$^2$ and add the Poisson noise. Finally, we transformed the photon fluence into CCD readings and added the dark current and the readout noise to form our multispectral datasets.

Finally, we reconstructed the source distribution in the mouse phantom from the multispectral datasets collected using our proposed system and the counterparts without any missing photons. The results based on our proposed data acquisition mode demonstrated that the positions of the reconstructed sources were accurately identified within a 1.5 mm error and the source power values reliably estimated within a 20% error, as shown in Figure 4. On the other hand, the

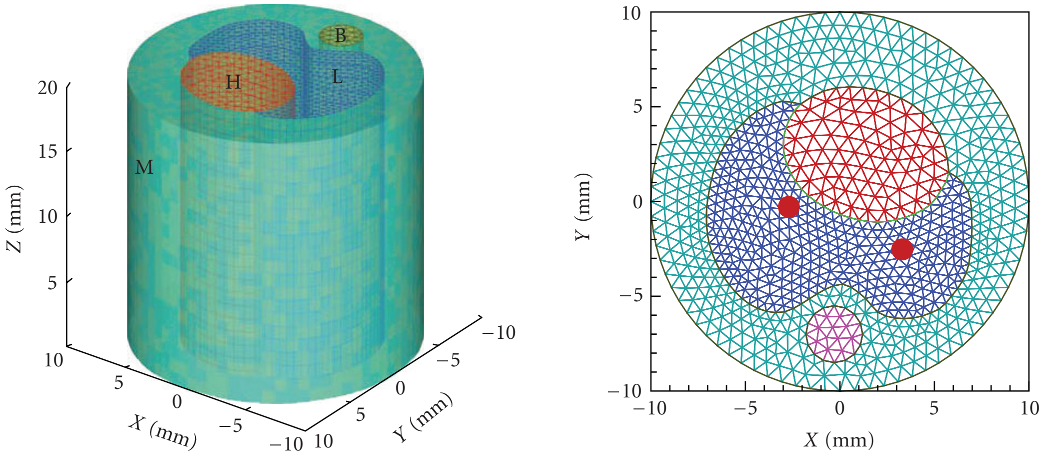

(a) (b)

Figure 3: Heterogeneous cylindrical mouse phantom of diameter 20 mm and height 20 mm. (a) The phantom containing four types of materials for muscle (M), lungs (L), heart (H), and bone (B), respectively, and (b) a transverse section of the phantom with the true source locations shown as the two red disks.

Table 1: Optical parameters used for the mouse chest phantom [17, 24].

| | Organ | | | | | | | |
|---|---|---|---|---|---|---|---|---|
| | Heart ($mm^{-1}$) | | Lung ($mm^{-1}$) | | Muscle ($mm^{-1}$) | | Bone ($mm^{-1}$) | |
| $\lambda$ | $\mu_a$ | $\mu_s'$ | $\mu_a$ | $\mu_s'$ | $\mu_a$ | $\mu_s'$ | $\mu_a$ | $\mu_s'$ |
| [500, 590] nm | 0.14 | 1.00 | 0.45 | 2.00 | 0.30 | 0.89 | 0.47 | 2.73 |
| [590, 625] nm | 0.11 | 1.10 | 0.35 | 2.30 | 0.23 | 1.00 | 0.35 | 3.84 |
| [625, 750] nm | 0.05 | 1.35 | 0.25 | 2.80 | 0.14 | 1.20 | 0.11 | 4.56 |

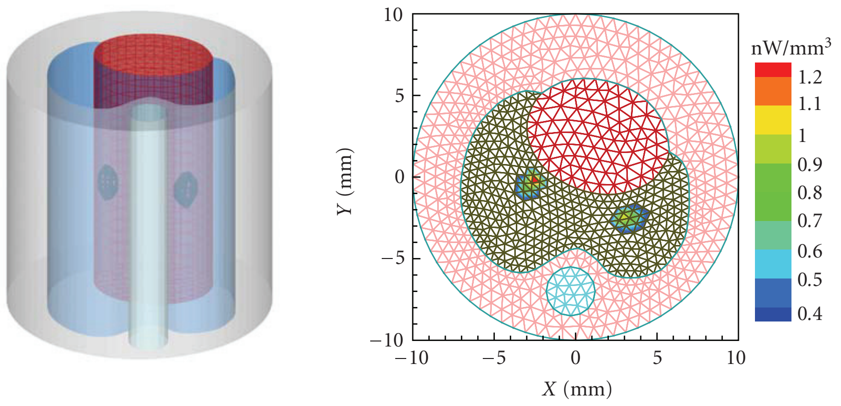

(a) (b)

Figure 4: Bioluminescent source reconstruction from datasets collected using our proposed system. (a) A volume rendering of the 3D bioluminescent source reconstruction, and (b) a transverse section through the reconstructed sources.

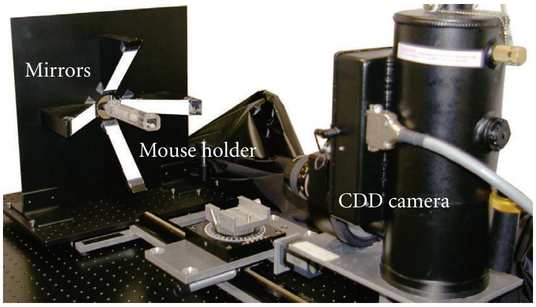

Figure 5: Prototype of a multiview system consisting of a multiview mirror system, a mouse holder, and a CCD camera. For illustration purpose, the four mirrors are colored in white.

counterparts based on the traditional sequential data acquisition mode indicated that the positions of the reconstructed sources were identified up to a 1.5 mm error and the source power values estimated up to a 16% error.

### 4.3. *Experiment results*

A prototype system with the multiview capability was developed to show the feasibility of our design. Figure 5 shows the main structure of the system without the rainbow mouse holder. Figure 6(a) shows the four bioluminescent views of a mouse captured by the system in the spectral band [500, 750] nm with 5-minute exposure time. Figures 6(b) and 6(c) are, respectively, the corresponding red box area in Figure 6(a) and the corresponding image captured using our original BLT system (sequential system). Both the images took the same exposure time and mapped to pseudocolor using the same method. Since the two systems had different fields of view (FOV), the corresponding areas did not have the same size in the images, and we had to match their sizes by scaling. Since the sequential system took the image after the multiview system, the multiview image looks brighter. Clearly, the two systems produced very similar signals.

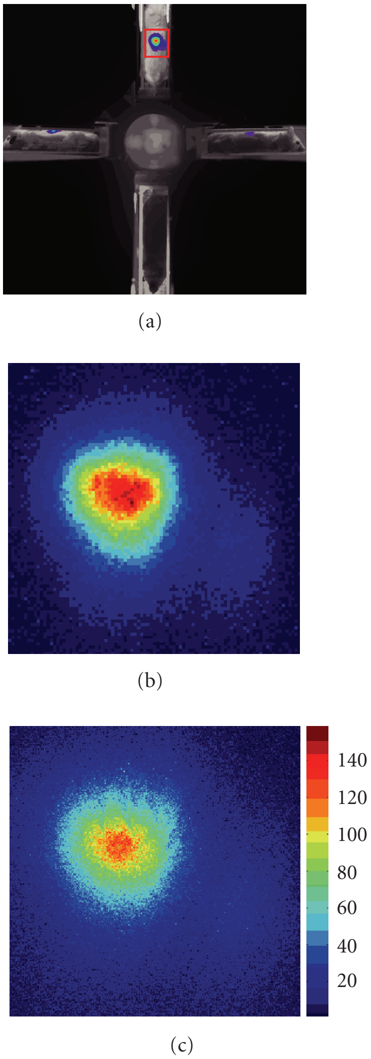

Figure 6: Bioluminescence images captured by our multiview system and the sequential system. (a) The four-view image of a mouse captured by the system in the spectral band [500, 750] nm for 5-minute exposure time, (b) the corresponding image delimited by the red box in (a), and (c) the corresponding image captured using our original BLT system (sequential system).

### 5. DISCUSSIONS AND CONCLUSION

Our system design dramatically improves the throughput of mouse studies. Although the bioluminescent signal is polychromatic, its variation is slow across the mouse body surface. Hence, the use of the rainbow mouse holder would not cause any significant information loss, as demonstrated in our SNR analysis, numerical and experimental studies. It is well known that the bioluminescence source intensity decays over time. A sequential system collects various views in each individual spectral band at different times. Thus, we need to compensate for the signal difference intrinsic to the sequential collection mode. On the other hand, using the multiview and multispectral system, we do not have this problem, every channel is collected simultaneously.

To improve the bioluminescence tomography results, using our proposed system we can capture more views by rotating the mirror system. For example, we can rotate the mirrors 45° to capture 8 views in total. Our system can be refined to have more than four mirrors surrounding the mouse to acquire more data, and even use a single mirror in the shape of a truncated cone with its narrower opening attached to the mounting plate and its side surface making 45° with respect to the mounting plate. To capture more photons from the mouse body surface, our system can be improved using a more complicated optical design in the same spirit of our current design. However, it is considered beyond the scope of this paper.

In conclusion, for the first time we have presented a simultaneous multiview and multispectral data collection system for BLT using four symmetrically arranged mirrors and a rainbow mouse holder. Our numerical and experimental results have demonstrated that bioluminescent signals collected using our new system can produce a similar BLT reconstruction quality while reducing the data acquisition time, as compared to that using the sequential data acquisition mode. We believe that our system design represents a major step forward in the development of BLT and its biomedical applications. Relevant in-vivo mouse results will be reported in the future.

### ACKNOWLEDGMENT

This work was supported by NIH/NIBIB EB001685.

### REFERENCES

[1] B. W. Rice, M. D. Cable, and M. B. Nelson, "In vivo imaging of light-emitting probes," *Journal of Biomedical Optics*, vol. 6, no. 4, pp. 432–440, 2001.

[2] C. H. Contag and M. H. Bachmann, "Advances in in vivo bioluminescence imaging of gene expression," *Annual Review of Biomedical Engineering*, vol. 4, pp. 235–260, 2002.

[3] V. Ntziachristos, J. Ripoll, L. V. Wang, and R. Weissleder, "Looking and listening to light: the evolution of whole-body photonic imaging," *Nature Biotechnology*, vol. 23, no. 3, pp. 313–320, 2005.

[4] G. Wang, E. A. Hoffman, and G. McLennan, "Systems and methods for bioluminescent CT reconstruction," Patent disclosure filled, July 2002; US provisional patent application filled, March 2003; US patent application filed, March 2004.

[5] G. Wang, et al., "Multi-spectral bioluminescence tomography methods and systems," Patent disclosure filed with University of Iowa Research Foundation, April 2004; provisional patent filed.

[6] G. Wang, D. Kumar, H. Shen, X. Qian, and W. Cong, "The Optical molecular tomography systems and methods for simultaneous acquisition of multi-view and multi-spectral data," Patent disclosure filed with University of Iowa Research Foundation, May 2006.

[7] G. Wang, E. A. Hoffman, G. McLennan, et al., "Development of the first bioluminescent CT scanner," *Radiology*, vol. 229(P), p. 566, 2003.

[8] G. Wang, Y. Li, and M. Jiang, "Uniqueness theorems in bioluminescence tomography," *Medical Physics*, vol. 31, no. 8, pp. 2289–2299, 2004.

[9] M. Jiang and G. Wang, "Image reconstruction for bioluminescence tomography," in *Developments in X-Ray Tomography IV*, vol. 5535 of *Proceedings of SPIE*, pp. 335–351, Denver, Colo, USA, August 2004.

[10] W. Cong, D. Kumar, Y. Liu, A. Cong, and G. Wang, "A practical method to determine the light source distribution in bioluminescent imaging," in *Developments in X-Ray Tomography IV*, vol. 5535 of *Proceedings of SPIE*, pp. 679–686, Denver, Colo, USA, August 2004.

[11] W. Cong, G. Wang, D. Kumar, et al., "Practical reconstruction method for bioluminescence tomography," *Optics Express*, vol. 13, no. 18, pp. 6756–6771, 2005.

[12] W. Cong and G. Wang, "Boundary integral method for bioluminescence tomography," *Journal of Biomedical Optics*, vol. 11, no. 2, Article ID 020503, 3 pages, 2006.

[13] W. Cong, D. Kumar, L. V. Wang, and G. Wang, "A Born-type approximation method for bioluminescence tomography," *Medical Physics*, vol. 33, no. 3, pp. 679–686, 2006.

[14] A. Cong and G. Wang, "Multispectral bioluminescence tomography: methodology and simulation," *International Journal of Biomedical Imaging*, vol. 2006, Article ID 57614, 7 pages, 2006.

[15] X. Gu, Q. Zhang, L. Larcom, and H. Jiang, "Three-dimensional bioluminescence tomography with model-based reconstruction," *Optics Express*, vol. 12, no. 17, pp. 3996–4000, 2004.

[16] C. Kuo, O. Coquoz, T. Troy, D. Zwarg, and B. Rice, "Bioluminescent tomography for in vivo localization and quantification of luminescent sources from a multiple-view imaging system," *Molecular Imaging*, vol. 4, p. 370, 2005.

[17] G. Alexandrakis, F. R. Rannou, and A. F. Chatziioannou, "Tomographic bioluminescence imaging by use of a combined optical-PET (OPET) system: a computer simulation feasibility study," *Physics in Medicine and Biology*, vol. 50, no. 17, pp. 4225–4241, 2005.

[18] A. J. Chaudhari, F. Darvas, J. R. Bading, et al., "Hyperspectral and multispectral bioluminescence optical tomography for small animal imaging," *Physics in Medicine and Biology*, vol. 50, no. 23, pp. 5421–5441, 2005.

[19] N. V. Slavine, M. A. Lewis, E. Richer, and P. P. Antich, "Iterative reconstruction method for light emitting sources based on the diffusion equation," *Medical Physics*, vol. 33, no. 1, pp. 61–68, 2006.

[20] H. Dehghani, S. C. Davis, S. Jiang, B. W. Pogue, K. D. Paulsen, and M. S. Patterson, "Spectrally resolved bioluminescence optical tomography," *Optics Letters*, vol. 31, no. 3, pp. 365–367, 2006.

[21] G. Wang, H. Shen, W. Cong, S. Zhao, and G. W. Wei, "Temperature-modulated bioluminescence tomography," *Optics Express*, vol. 14, no. 17, pp. 7852–7871, 2006.

[22] H. Zhao, T. C. Doyle, O. Coquoz, F. Kalish, B. W. Rice, and C. H. Contag, "Emission spectra of bioluminescent reporters and interaction with mammalian tissue determine the sensitivity of detection in vivo," *Journal of Biomedical Optics*, vol. 10, no. 4, Article ID 041210, 9 pages, 2005.

[23] A. Ishimaru, *Wave Propagation and Scattering in Random Media*, Oxford University Press, Oxford, UK, 1997.

[24] F. Natterer and F. Wüng, *Mathematical Methods in Image Reconstruction*, Society for Industrial and Applied Mathematics, Philadelphia, Pa, USA, 2001.

[25] http://omlc.ogi.edu/spectra/hemoglobin/index.html.

[26] V. Tuchin, *Tissue Optics: Light Scattering Methods and Instruments for Medical Diagnosis*, SPIE-International Society for Optical Engine, Bellingham, Wash, USA, 2000.

[27] W. F. Cheong, S. A. Prahl, and A. J. Welch, "A review of the optical properties of biological tissues," *IEEE Journal of Quantum Electronics*, vol. 26, no. 12, pp. 2166–2185, 1990.

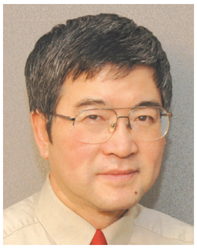

**Ge Wang** received the B.E. degree in electrical engineering from Xidian University, Xian, China, in 1982, the M.S. degree in remote sensing from the Graduate School of Academia Sinica, Beijing, China, in 1985, and the M.S. and Ph.D. degrees in electrical and computer engineering from the State University of New York, Buffalo, in 1991 and 1992. He was an Instructor and Assistant Professor with the Department of Electrical Engineering, Graduate School of Academia Sinica, in 1984–1988, and Instructor and Assistant Professor with Mallinckrodt

Institute of Radiology, Washington University, St. Louis, Mo, in 1992–1996. He was an Associate Professor with the University of Iowa in 1997–2002. Currently, he is a Professor with the Departments of Radiology, Biomedical Engineering, Mathematics, Civil Engineering, and Electrical and Computer Engineering, and Director of the Center for X-Ray and Optical Tomography, University of Iowa. His interests include computed tomography, bioluminescence tomography, and systems biomedicine. He has published over 300 journal articles and conference papers, including the first paper on spiral/helical cone-beam CT and the first paper on bioluminescence tomography. He is the Editor-in-Chief for the International Journal of Biomedical Imaging, and an Associate Editor for the IEEE Transactions on Medical Imaging and the Journal of Medical Physics. He is an IEEE Fellow and an AIMBE Fellow. He is also recognized by a number of awards for academic achievements.

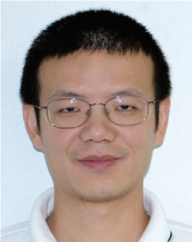

**Haiou Shen** received the B.E. and M.E. degrees in electrical engineering from Wuhan University, Wuhan, China, in 1995 and 1998. He is currently a Ph.D. student in the Department of Computer Science at the University of Iowa. He is currently working as a research assistant in the BLT Research Group, Department of Radiology at the University of Iowa. His research interests include satisfiability problem and bioluminescence tomography.

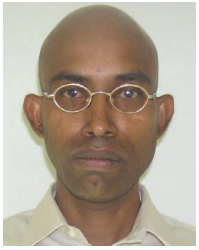

**Kumar Durairaj** received his Ph.D. degree in biomedical engineering from IIT-Madras, in 2003. His interest includes laser instrumentation, biomedical optics, and image processing. At present, he is working as a Professor in the Department of Electronics and Communication Engineering, Periyar Maniammai College of Technology for Women, Vallam Thanjavur, India.

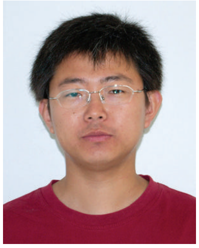

**Xin Qian** received his B.E. degree from Tianjin University, Tianjin, China, in 2001, and Ph.D. degree in fiber optics from Bournemouth University, Southampton, UK, in 2005. He is presently working as a Post Doctor with the BLT Research Group at the University of Iowa, USA. His research interests include biomedical imaging and fiber-based optical devices.

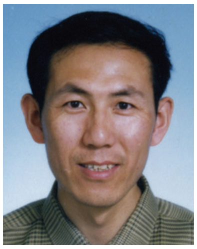

**Wenxiang Cong** received his B.S. degree in mathematic from Heilongjiang University, Harbin, China, in 1982, M.S. degree in applied mathematic from Harbin Institute of Technology, Harbin, China, in 1988, and Ph.D. degree in optical imaging from Beijing University of Science and Technology, Beijing, China, in 1998. Currently, he is a Research Scientist with the Bioluminescence Tomography Laboratory, Department of Radiology, University of Iowa, Iowa City, Iowa, USA. His research interests include biomedical imaging, optical tomography and bioluminescence tomography. He authored or coauthored 30 journal papers and 10 conference proceedings. He is a Member of the Optical Society of America.

